# Spread of *Streptococcus suis* Sequence Type 7, China

**DOI:** 10.3201/eid1405.070437

**Published:** 2008-05

**Authors:** Changyun Ye, Xuemei Bai, Ji Zhang, Huaiqi Jing, Han Zheng, Huamao Du, Zhigang Cui, Shouying Zhang, Dong Jin, Yanmei Xu, Yanwen Xiong, Ailan Zhao, Xia Luo, Qiangzheng Sun, Marcelo Gottschalk, Jianguo Xu

**Affiliations:** * State Key Laboratory for Infectious Disease Prevention and Control, Beijing, People’s Republic of China; † National Institute for Communicable Disease Control and Prevention, Changping, People’s Republic of China; ‡ Université de Montréal, Montréal, Québec, Canada; 1 These authors contributed equally to this article.

**Keywords:** *Streptococcus suis*, multilocus sequence typing (MLST), tetracycline resistance, transposon, dispatch

## Abstract

*Streptococcus suis* sequence type (ST) 7 has been spreading throughout China. To determine events associated with its emergence, we tested 114 isolates. In all 106 ST7 strains responsible for human outbreaks and sporadic infections, the tetracycline-resistance gene, *tetM,* was detected on the conjugative transposon Tn*916.* Horizontal transmission of *tetM* is suspected.

A large outbreak of *Streptococcus suis* serotype 2 infection emerged in the summer of 2005 in Sichuan Province, People’s Republic of China, and resulted in 215 cases and 38 deaths among humans (*1*). Sporadic infections were identified in 4 other provinces. A smaller, previously overlooked, outbreak occurred in Jiangsu Province in 1998; 25 cases and 14 deaths were reported (*1*,*2*). The causative agent of the Sichuan and Jiangsu outbreaks was identified as a clone of *S. suis* sequence type (ST) 7 (*3*). ST7 was first identified in 1996 in a patient with meningitis in Hong Kong and later caused the 1998 outbreak in Jiangsu; it spread further to cause the largest outbreak in Sichuan in 2005 (*3*,*4*). The spread of *S. suis* ST7 across China underscores the need to better understand the genetic and ecologic events associated with its emergence as an important pathogen in humans.

## The Study

Using the MICroSTREP Plus system (Dade Behring, Deerfield, IL, USA), we tested 114 ST7 isolates from China and found that all isolates were resistant to tetracycline and susceptible to 12 of 13 antimicrobial drugs. Of these 114, 6 were isolated in 2006, 84 were from human patients and 8 from diseased pigs in the 2005 Sichuan outbreak, 7 were from sporadic human cases and 3 from diseased pigs in other provinces in 2005, and 4 were from human patients and 2 from diseased pigs in the 1998 Jiangsu outbreak (Table 1). The isolates were susceptible to penicillin, ampicillin, cefotaxime, ceftriaxone, cefepime, meropenem, levofloxacin, chloramphenicol, erythromycin, azithromycin, clindamycin, and vancomycin. In contrast, 7 of 12 *S. suis* serotype 2 strains from other countries and 18 of 34 serotype reference strains were resistant to tetracycline; 3 tetracycline-resistant strains were also resistant to erythromycin, azithromycin, and clindamycin.

**Table 1:** Source, serotype, sequence type, and tetracycline-resistant genes in *Streptococcus suis* strains*

No. strains	Source (no.)	Place of isolation	Year of isolation (no.)	Serotype (no.)	ST (no.)	*tet* gene (no. positive)	Tn*916* (no.)	Virulence genes (no.)			
*cps2j*	*mrp*	*sly*	*ef*
98 outbreak-associated ST7 strains in China											
84	Human patients	Sichuan, China	2005	2	ST7	*tetM* (84)	Intact (84)	+ (84)	+ (84)	+ (84)	+ (84)
8	Diseased pigs	Sichuan, China	2005	2	ST7	*tetM* (8)	Intact (8)	+ (8)	+ (8)	+ (8)	+ (8)
4	Human patients	Jiangsu, China	1998	4	ST7	*tetM* (4)	Intact (4)	+ (4)	+ (4)	+ (4)	+ (4)
2	Diseased pigs	Jiangsu, China	1998	2	ST7	*tetM* (2)	Intact (2)	+ (2)	+ (2)	+ (2)	+ (2)
8 ST7 strains isolated from sporadic cases in China											
5	Human patients	6 provinces, China	2005 (2) 2006 (3)	2	ST7	*tetM* (5)	Intact (5)	+ (5)	+ (5)	+ (5)	+ (5)
3	Diseased pigs	Jiangxi, China	2005	2	ST7	*tetM* (3)	Intact (3)	+ 3)	+ (3)	+ (3)	+ (3)
7 ST1 and 1 untypeable strain isolated from sporadic cases in China											
5	Human patients	Guizhou, Guangxi (4)	2005	2 (4) 14	ST1	*tetM* (4)	Intact (4)	+ (4)	+ (5)	+ (5)	+ (5)
3	Human patients	3 provinces	2006	2	ST1 (2) UT	*tetO* (2) *tetM*	Intact (1)	+ (3)	+ (3)	+ (3)	+ (3)
12 serotype 2 strains from other countries											
5	Human patients	Netherlands (2), France (3)	NA	2	ST1	*tetO* (3)		+ (5)	+ (5)	+ (5)	+ (4)
3	Diseased pigs	Netherlands, France, England	NA	2	ST1			+ (3)	+ (3)	+ (3)	+ (2)
4	Human patients (2), healthy pigs, diseased pigs	Canada (3), England	NA	2	ST25	*tetO* (4)		+ (4)	–	–	–
34 serotype reference strains											
1	Human patients	Netherlands	NA	14	ST6			–	–	+	–
1	Diseased pig	Denmark	NA	13	ST71	*tetM*	Intact	–	–	+	–
25	Diseased pigs	Canada (11), Denmark (5), Netherlands (9)	NA	1/2, 2–12, 15–16, 22–30, 32, 34	ST1, ST35, ST53–55, ST68, ST69, ST72–73, ST75, ST77–78, ST80–82, ST87, ST91–92, UT (7)	*tetO* (11)		+ (2)	+ (2)	+ (7)	+ (1)
2	Diseased calves	Canada, United States	NA	20, 31	ST70 (1), UT (1)	*tetO* (1)		–	–	–	–
1	Diseased lamb	Canada	NA	33	UT			–	–	–	–
4	Healthy pigs	Canada	NA	17–19, 21	ST76 (2), ST79, UT	*tetO* (4)		–	–	+ (2)	–

*ST, sequence type; *tet*, tetracycline; UT, untypeable by multilocus sequence typing; NA, not available.

Multilocus sequence typing analysis showed that of the 114 isolates from China, 106 were typed as ST7: 98 from the Sichuan and Jiangsu outbreaks, 5 from sporadic infections in other provinces in 2005 and 2006, and 3 from diseased pigs from other provinces in 2005. Of the other 8 isolates from sporadic cases in 2005 and 2006, 7 were ST1 and 1 was untypeable. Of the 12 serotype 2 strains from other countries, 8 were ST1 and 4 were ST25. Of the 34 serotype reference strains, serotype 2 strain R735 was ST1, 10 serotypes were untypeable, and 22 STs were identified as ST6 (serotypes 17 and 19), ST35, ST53-55, ST68-73, ST75-82, ST87, or ST91-2. Serotype 17 and 19 strains were identified as ST76 (Table 1) (*5*,*6*).

PCR was used to screen all isolates for tetracycline resistance genes; primers specific for *tetABCDEGKLMOQS* were used (*7*,*8*). Of the 114 tetracycline-resistant isolates from China, 111 (all 106 ST7 strains and 5 of 7 ST1 strains) harbored the *tetM* gene. The *tetO* gene was carried by 1 ST1 and 1 sequence-untypeable strain. All 7 tetracycline-resistant serotype 2 strains from other countries and 16 of 18 tetracycline-resistant strains in the 34 reference serotypes carried the *tetO* gene (Table 1) (*5*,*6*). The only other *tetM*-positive strain was from serotype 13, an ST71 isolated from a diseased pig in Denmark (Table 1). The PCR results were confirmed by sequencing the PCR-synthesized fragments.

**Figure 1:**
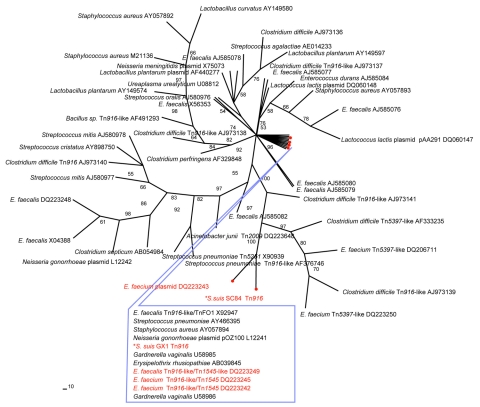
Phylogenetic relationship of the *tetM* sequences of *Streptococcus suis.* An unrooted maximum-parsimony tree was based on multiple aligned partial *tetM* sequences of 2 *S. suis* (asterisk) and 53 reference sequences retrieved from GenBank. The alignment length for the analysis was 1,415 bp. If available, the designation of the *tetM*-carrying plasmid or transposon is indicated, followed by the GenBank accession number. Percent bootstrap support at each internal node was based on 200 replicate trees. The sequences of known pig origin are marked in red.

To further characterize the *tetM* genes, the open reading frame (ORF) was completely sequenced by using 16 selected strains: 9 isolates from humans and 1 from a pig from the Sichuan outbreak; 3 from sporadic infections in Guangxi, Jiangsu, and Guangdong; 1 from a diseased pig in Jiangxi Province in 2005; and 2 from the Jiangsu outbreak in 1998 (Table 1). Sequence alignments showed 2 groups (GenBank accession nos. EF101931, EF016118). The first group comprised 15 of the 16 isolates typed as ST7 (*3*). The second group had only 1 isolate, GX1, typed as ST1 (*3*). The sequences of *tetM* gene for ST7 (strain SC84) and ST1 (GX1) were 1,920 and 1,917 bp, respectively, with 90 nt variations between the 2 sequences leading to 32 aa changes. Comparison of the 53 *tetM* sequences with those from public databases showed that the *tetM* of *S. suis* SC84 was most related to *Enterococcus faecium* isolate 9830470-4 plasmid pYA470-4 (DQ223243) (*7*). The *tetM* sequence of *S. suis* ST1 strain GX1 was most closely related to *S. pneumoniae* Tn916-like/Tn2009 (AY466395) and to *Gardnerella vaginali* (U58986) (Figure 1) (*9*).

Because the gene *tetM* is reported to be associated with transposon Tn*916*, we designed 24 pairs of primers targeting its 24 ORFs based on published Tn*916* sequences (Table 2). The complete sequence of Tn*916* from SC84 was obtained by sequencing the PCR-synthesized fragments. Between Tn*916* of SC84 and plasmid pYA470-4 of *E. faecium*, we observed 133 nt variations, 91 of which were in the *tetM* gene, 3 in the integrase gene, 1 in excisionase gene, and 38 in 10 additional ORFs. PCR showed that 111 isolates from China and 1 from Denmark have intact Tn*916* (Table 1).

**Table 2:** Primers used to detect *tet* genes and conjugative transposon Tn*916* in Streptococcus suis*

Gene	Primers (5′ → 3′)	Product size, bp	Annealing temperature, °C
*tetA*	GCTACATCCTGCTTGCCTTC; CATAGATCGCCGTGAAGAGG	210	55
*tetB*	TTGGTTAGGGGCAAGTTTTG; GTAATGGGCCAATAACACCG	659	55
*tetC*	CTTGAGAGCCTTCAACCCAG; ATGGTCGTCATCTACCTGCC	418	55
*tetD*	AAACCATTACGGCATTCTGC; GACCGGATACACCATCCATC	787	55
*tetE*	AAACCACATCCTCCATACGC; AAATAGGCCACAACCGTCAG	278	55
*tetG*	GCTCGGTGGTATCTCTGCTC; AGCAACAGAATCGGGAACAC	468	55
*tetK*	TCGATAGGAACAGCAGTA; CAGCAGATCCTACTCCTT	169	55
*tetL*	TCGTTAGCGTGCTGTCATTC; GTATCCCACCAATGTAGCCG	267	55
*tetM*	GTGGACAAAGGTACAACGAG; CGGTAAAGTTCGTCACACAC	406	55
*tetO*	AACTTAGGCATTCTGGCTCAC; TCCCACTGTTCCATATCGTCA	515	55
*tetQ*	TTATACTTCCTCCGGCATCG; ATCGGTTCGAGAATGTCCAC	904	55
*tetS*	CATAGACAAGCCGTTGACC; ATGTTTTTGGAACGCCAGAG	667	55
ORF 24	ATGAGGTGTCTATTTTTTTA; TTATTGGCTGAATGAATGTT	120	52
ORF 23	AATTTGTGATTCCCAACATG; CGTCAGCATGTAAAAGGTAA	315	52
ORF 22	ATGATGAGATTAGCAAATGG; CTATTTGTCTTGTGTCGGTT	387	52
ORF 21	TTTCATTTTACGATAGCGTC; GTCGCCTGCGTGGACTGTCT	1,308	55
ORF 20	ATGCTGTTTGATTATGTAAG; TTATTTTTTTGTTGTTATCA	990	52
ORF 19	ATGAATTTTGGACAAAACCT; TTAAGCACCAATAATGCGAT	222	52
ORF 18	TTTAGGCAAATACAATGAGG; GATTGGTTGAGATAAACGTT	443	56
ORF 17	ATGGGATTTTTGAAATCGTC; TTAATTGGATATGCCATAAA	507	52
ORF 16	ATGGCATATCCAATTAAATA; TTACACCTCTTTTCGCACAG	2,448	52
ORF 15	ATGTGAAACCATCAATAGTA; TCATCTGAAAATAAAATGGC	2,265	52
ORF 14	ATGAAGTTGAAAACTTTAGT; TCATTGTTTGATTCGTCCTG	1,002	52
ORF 13	AGAAAAACAGATACCAAAGG; CGTTCTTTTCAAGTACCAAA	860	54
ORF 12-*tetM*	ATGCTTTGTATGCCTATGGT; CTAAGTTATTTTATTGAACA	2022	54
ORF 5–10	ATTATAAACTACAAGTGGAT; TTCGTTTAATCTGAATACGA	2233	52
*Xis*-Tn	ATGAAGCAGACTGACATTCC; TTCGTTTAATCTGAATACGA	204	52
*Int*-Tn	GACTGGAGAGAGCCAACGAA; CATCATGCCGTTGTAATCAC	925	54

*tet, tetracycline; ORF, open reading frame.

The most recognized virulence genes of *S. suis,* including *mrp*, *sly*, and *ef,* were detected by PCR in all 114 Chinese isolates tested in this study. Of the 12 serotype 2 strains from other countries, 6 of 8 ST1 strains were positive for all 3 virulence genes. However, none of the 4 ST25 strains tested positive (Table 1). Of the 34 serotype reference strains, serotype 2 strain R735 was positive for *mrp* and *sly*. The *sly* gene was detected in 10 reference strains that were typed serotype 1/2 as untypeable, serotype 2 as ST1, serotype 4 as ST54, 5 as ST53, 7 as untypeable, 13 as ST71, 14 as ST6, 16 as ST73, 17 as ST76, and 18 as ST79 (Table 1).

**Figure 2:**
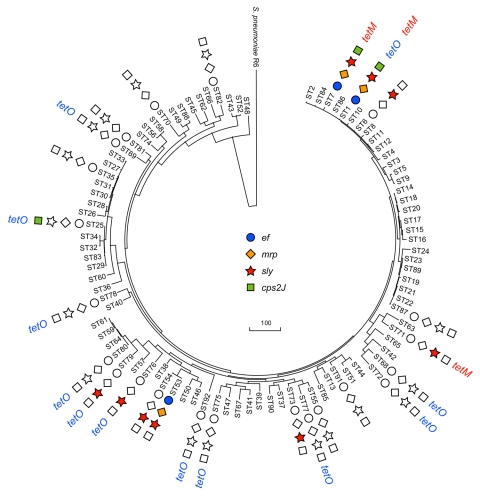
Horizontal transfer events of virulence genes and conjugative transposon Tn*916* with *tetM* in *Streptococcus suis* sequence types. The rooted maximum-parsimony tree was based on the concatenated sequences of 7 housekeeping genes used for multilocus sequence typing analysis of *S. suis* by using S. *pneumoniae* R6 as the outgroup. Virulence genes acquired by strains of various sequence types were from this study and other published papers. The colored labels indicate positive detection; uncolored labels indicate negative results for the virulence gene; no label indicates that the strain was not available for testing. The scale bar indicates a branch length corresponding to 100 character-state changes.

To determine the significance of horizontal gene transfer of Tn*916* with the *tetM* and virulence genes, we constructed a rooted phylogenetic tree by using the maximum-parsimony method. The sequence of *S. pneumoniae* R6 was chosen as the outgroup that is closely related to *S. suis* (*10*). The data suggest *S. suis* ST7 evolved originally from ST1 and ST48. The horizontal transfer of tested virulence genes and *tetM* occurred in various stages of the evolution of *S. suis* and played a major role in the emergence of ST1 and ST7 (Figure 2).

## Conclusions

We report that *S. suis* ST7 was responsible for 2 large outbreaks and sporadic infections in several provinces of China and has recently acquired the tetracycline resistance gene, *tetM,* associated with the conjugative transposon Tn*916* (*3*,*7*). Horizontal transfer of Tn*916* with the *tetM* gene occurred in at least 3 STs located at various stages in the constructed phylogenetic tree and played a central role in the evolution of the epidemic *S. suis* ST7 clone. All 3 virulence genes tested in this study were shown to be transferred horizontally (*11*–*13*).

Our data support the contention that Tn*916* with *tetM* acts as an important selective factor that provides considerable advantages for the clone emergence and spread of *S. suis* ST7 (*3*). The widespread use of tetracycline in swine feed could provide the selective pressure for clone amplification and spreading, thus contributing to the outbreak of *S. suis* ST7 through Tn*916* (*14*). The countrywide spread of *S. suis* ST7-*tetM* represents a model of selective pressure leading to the emergence of a bacterium as a virulent pathogen in humans. The case of *S. suis* ST7 is a sign that pathogens present in food animals can result in substantial public health problems if no action is taken to prevent the indiscriminate use of antimicrobial drugs in animal feed (*15*).
